# Applying machine-learning models to differentiate benign and malignant thyroid nodules classified as C-TIRADS 4 based on 2D-ultrasound combined with five contrast-enhanced ultrasound key frames

**DOI:** 10.3389/fendo.2024.1299686

**Published:** 2024-04-03

**Authors:** Jia-hui Chen, Yu-Qing Zhang, Tian-tong Zhu, Qian Zhang, Ao-xue Zhao, Ying Huang

**Affiliations:** Department of Ultrasound, Shengjing Hospital of China Medical University, Shenyang, China

**Keywords:** thyroid nodules, ultrasound, contrast-enhanced ultrasound, machine learning, radiomics features, key frames, radiologists

## Abstract

**Objectives:**

To apply machine learning to extract radiomics features from thyroid two-dimensional ultrasound (2D-US) combined with contrast-enhanced ultrasound (CEUS) images to classify and predict benign and malignant thyroid nodules, classified according to the Chinese version of the thyroid imaging reporting and data system (C-TIRADS) as category 4.

**Materials and methods:**

This retrospective study included 313 pathologically diagnosed thyroid nodules (203 malignant and 110 benign). Two 2D-US images and five CEUS key frames (“2^nd^ second after the arrival time” frame, “time to peak” frame, “2^nd^ second after peak” frame, “first-flash” frame, and “second-flash” frame) were selected to manually label the region of interest using the “Labelme” tool. A total of 7 images of each nodule and their annotates were imported into the Darwin Research Platform for radiomics analysis. The datasets were randomly split into training and test cohorts in a 9:1 ratio. Six classifiers, namely, support vector machine, logistic regression, decision tree, random forest (RF), gradient boosting decision tree and extreme gradient boosting, were used to construct and test the models. Performance was evaluated using a receiver operating characteristic curve analysis. The area under the curve (AUC), sensitivity, specificity, positive predictive value (PPV), negative predictive value (NPV), accuracy (ACC), and F1-score were calculated. One junior radiologist and one senior radiologist reviewed the 2D-US image and CEUS videos of each nodule and made a diagnosis. We then compared their AUC and ACC with those of our best model.

**Results:**

The AUC of the diagnosis of US, CEUS and US combined CEUS by junior radiologist and senior radiologist were 0.755, 0.750, 0.784, 0.800, 0.873, 0.890, respectively. The RF classifier performed better than the other five, with an AUC of 1 for the training cohort and 0.94 (95% confidence interval 0.88–1) for the test cohort. The sensitivity, specificity, accuracy, PPV, NPV, and F1-score of the RF model in the test cohort were 0.82, 0.93, 0.90, 0.85, 0.92, and 0.84, respectively. The RF model with 2D-US combined with CEUS key frames achieved equivalent performance as the senior radiologist (AUC: 0.94 vs. 0.92, *P* = 0.798; ACC: 0.90 vs. 0.92) and outperformed the junior radiologist (AUC: 0.94 vs. 0.80, *P* = 0.039, ACC: 0.90 vs. 0.81) in the test cohort.

**Conclusions:**

Our model, based on 2D-US and CEUS key frames radiomics features, had good diagnostic efficacy for thyroid nodules, which are classified as C-TIRADS 4. It shows promising potential in assisting less experienced junior radiologists.

## 1 Introduction

Thyroid nodules are a common clinical condition. In recent decades, the use of high-resolution ultrasound has rapidly increased worldwide (1, 2). The detection rate of thyroid nodules can reach 67%; however, only 5–15% of them are malignant (3, 4). In clinical practice, many patients suffer some complications after surgical thyroidectomy (5, 6). Moreover, the status quo of overdiagnosis and overtreatment has added unnecessary burdens to patients. In 2020, Chinese experts developed the Chinese version of the thyroid imaging reporting and data system (C-TIRADS) to evaluate the characteristics of thyroid nodules, providing a more practical and concise tool for daily clinical practice (7). Most nodules classified as C-TIRADS 3 or 5 can be quickly distinguished accurately using two-dimensional ultrasound (2D-US) alone; however, there is a wide range of malignancy rates among thyroid nodules classified as C-TIRADS 4 (2–90%). Moreover, some hypoechoic Hashimoto nodules with blurred margins can be classified as C-TIRADS 4 (8). and mummified nodules with internal necrotic components may also exhibit marked hypoechogenicity (9). Distinguishing these from malignant nodules poses challenges, leading to the low specificity of 2D-US and warranting fine needle aspiration (FNA), an invasive procedure (2). Thus, there is a need to explore new methods for a more precise diagnosis of thyroid nodules which are classified as C-TIRADS 4.

Contrast-enhanced ultrasound (CEUS), which describes focal microcirculation perfusion status by distinguishing acoustic features of tissue backgrounds, plays an essential role in the diagnosis of thyroid nodules and differentiation of necrotic benign nodules from malignant ones to avoid FNA procedures (10). Additionally, CEUS is utilized in the field of interventional ultrasonography, which includes assisting biopsy and FNA procedures and estimating therapeutic conditions after ablation (11, 12). Despite not being recommended as part of the guidelines for diagnosing thyroid nodules, numerous studies have demonstrated that CEUS exhibits a sensitivity and specificity of discriminating malignant nodules from benign nodules that could reach 0.87 and 0.83, respectively (13, 14). The consensus on the qualitative and quantitative analysis of CEUS recommends that malignant characteristics include later wash-in, heterogeneous hypoenhancement, earlier wash-out, and centripetal perfusion (15–17). Machine learning (ML) is an algorithm based on representational learning of data, except for computer vision, natural language processing, and speech recognition, and has played a prominent role in the medical field (18–21). ML can significantly limit interobserver variations (22). With the rapid development of artificial intelligence (AI), radiomics has recently attracted the attention of researchers. Radiomics can transform pixels in medical images into high-dimensional features and quantitative data that can be calculated, which could show intratumor heterogeneity and texture features (23, 24). ML algorithms can be used to develop predictive models and calculate their performances. In the field of thyroid nodules, ML is mostly based on 2D-US images, with an accuracy (ACC) of approximately 0.88–0.92 (25, 26). To our knowledge, only two studies have used CEUS images to build AI models for diagnosing thyroid nodules (27, 28). Wan et al. used deep learning (DL) to build a diagnostic model based on dynamic CEUS video and obtained relatively high performance (27). Guo et al. used logistic regression to build ML models based on US and CEUS features, while only included a single frame of CEUS images (28). Our study aimed to explore the useful information of CEUS images for diagnosing C- TIRADS 4 thyroid nodules. Herein, we combined 2D-US with five CEUS key frames as an import for further radiomics feature extraction and ML model development, aimed at examining the value of ML model based on 2D-US and CEUS key frames in the differential diagnosis of benign and malignant nodules which are classified as C- TIRADS 4.

## 2 Materials and methods

### 2.1 Patients

This retrospective study was conducted between September 2019 and February 2023. Data from 313 thyroid nodules in 300 patients which underwent FNA or thyroid surgery at our hospital were included in this study. The inclusion criteria were: (1) patients aged ≥18 years; (2) nodule classified as C-TIRADS 4 (with at least one malignant sign); (3) some suspicious malignant nodules that needed CEUS examination to exclude mummified nodules before FNA, and some cystic-solid nodules which were classified as C-TIRADS 3 but the most component were the solid and were eccentric distribution; (4) CEUS examination procedures that contained “double-flash” at 40s and 60s, respectively; and (5) patients who signed an informed consent form and obtain pathological results of thyroid nodules after the CEUS examination. The exclusion criteria were: (1) allergy to any of the components in the ultrasound contrast agent; (2) nodules with macrocalcification during B-mode ultrasound examination; (3) FNA pathological results incomplete or categorized as Bethesda I, III, and IV; and (4) CEUS videos with severe motion. The patients were separated at a ratio of 9:1. Our study was approved by Medical Ethics Committee of Shengjing Hospital of China Medical University (2023PS967K). The PASS.15 software (NCSS LLC, Kaysville, UT, USA) was used to calculate the sample size, with parameters set to ensure the power of 0.90 and level α was set at bilateral 0.05. Based on our expected results, the receiver operating characteristic (ROC) curve was set to 0.90. The false-positive rate was limited from 0 to 1. The group allocation was set at 2. The number of nodules included in the training cohort was 144 in the malignant group and 72 in the benign group (total = 216), with an additional 10% for dropouts. Hence, the final result was 158 and 80 nodules in the malignant and benign groups, respectively (total = 238).

### 2.2 US, CEUS examinations and images selection

An L14-3U transducer (frequency: 3–9 MHz) from the Resona 9 device (Mindray, Shenzhen, China) and an L12-5 transducer (frequency: 5–12 MHz) from the iU22 device (Philips, Amsterdam, The Netherlands) were used. 2D-US was performed by two radiologists, one with 3 years of experience in thyroid ultrasound and the other with >10 years of experience in thyroid ultrasound. We measured the thyroid size, nodule numbers, nodule size, nodule location, component, echogenicity, shape, margin, and the presence or absence of Hashimoto’s background and microcalcification. We then recorded following the C-TIRADS guidelines. In patients with multiple nodules, the ones most suspicious for malignancy were selected for observation and subsequent CEUS examination. The C-TIRADS classification was recorded, and nodules with inconsistent C-TIRADS results were reevaluated and decided upon. Subsequently, CEUS was performed by an experienced radiologist, who then selected the largest section of the nodule, including the surrounding normal thyroid tissue. The mechanical index was set to 0.06–0.08, and the gain, depth, acoustic window, and focal zone were adjusted. The probe stabilized, and the CEUS mode was initiated. For this procedure, 59 mg of contrast agent (SonoVue; Bracco, Milan, Italy) was mixed with 5 mL of saline to prepare a suspension. The suspension (1.5 mL) was injected rapidly through the superficial vein of the elbow, followed by a 5 mL saline flush. The timer was started simultaneously with the time of injection. The term “flash” means when the microbubbles had been blown up, the remaining microbubbles would reperfuse after the “flash” without the bolus’s influence, making good efforts to observe reperfusion status. The radiologist pressed the contrast agent click-button in the 40^th^ and 60^th^ seconds, defined as “first-flash” and “second-flash,” respectively. The entire dynamic recording lasted 80 seconds and was recorded in “AVI” format. Two experienced radiologists immediately diagnosed patients. CEUS observation parameters, including wash-in pattern (earlier, synchronous, and later), enhanced intensity (hypo-, iso-, and hyperintensity), enhanced homogeneity (homo- and heterogeneous), enhanced method (centripetal and centrifugal), and wash-out pattern (earlier, synchronous, and later), were recorded. The nodules with inconsistent results were examined and discussed. According to the previous studies (17, 29–32), nodules with “later wash-in”, “heterogeneous hypointensity”, “centripetal enhancement” and “earlier wash-out” were malignant parameters for thyroid nodules. In our study, we defined nodules with at least two of the among parameters as malignant nodules, the others were defined as benign nodules.

Furthermore, the nodule’s largest transverse and longitudinal sections were selected in 2D-US after rotating the probe 90° clockwise. Regarding CEUS, the perfusion of the contrast agents gradually changes with changes in brightness during CEUS examinations, which could reveal the blood supply of the nodule. Many previous studies have also suggested wash-in or -out patterns of contrast agents, and the enhanced intensity in the nodule area compared to the surrounding normal thyroid tissues was the most helpful parameter for diagnosing malignant nodules (11, 31, 33, 34). The “double-flash,” identified as a new CEUS quantitative parameter in our previous study, indicated that the diagnostic accuracy in distinguishing malignant and benign thyroid nodules could reach 88.4% (24). Therefore, based on these principles and results, five CEUS key frames were finally selected: the “2^nd^ second after the arrival time” frame, “time to peak” frame, “2^nd^ second after peak” frame, “first-flash” frame, and “second-flash” frame.

### 2.3 Nodule segmentation

**Figure 1:**
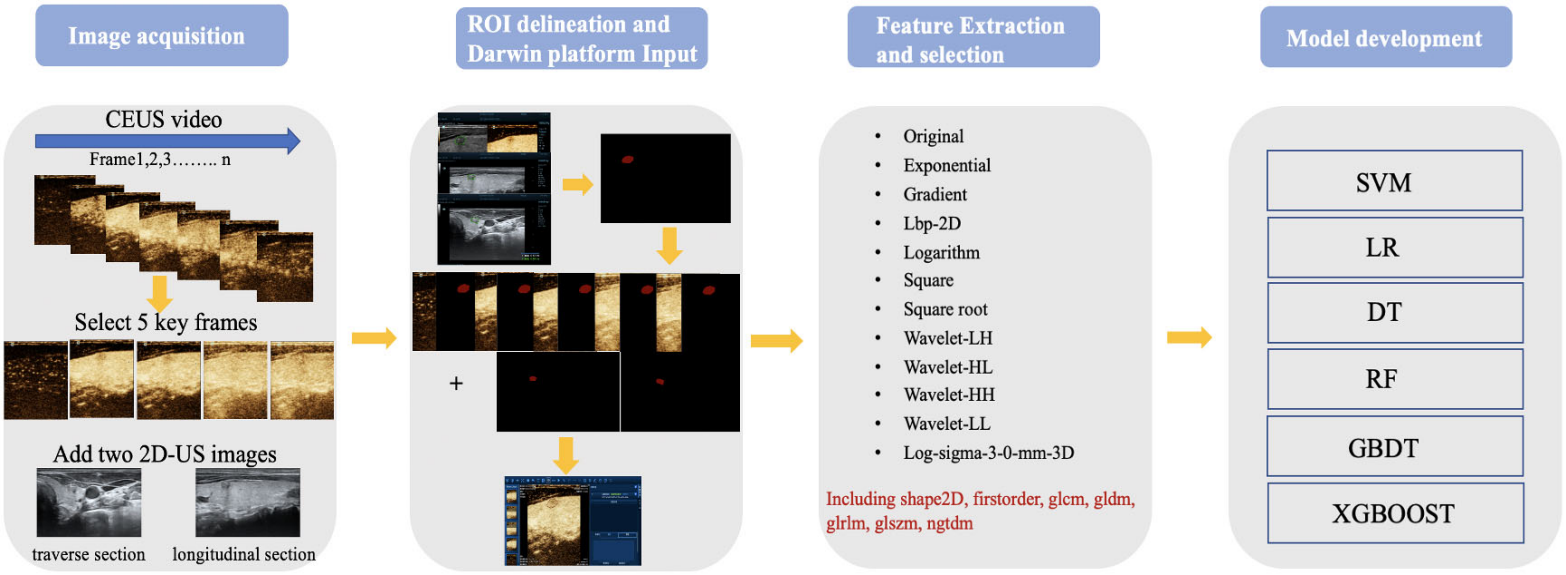
Workflow of image acquisition.

The 80-second CEUS video of each patient was converted to 1120 images (14 images every second) using Python code. One radiologist (with 3 years of CEUS experience) browsed the images and found five key CEUS frames. The radiologist manually delineated the boundary of the region of interest (ROI) on seven images (two from 2D-US and five from CEUS key frames) using “Labelme” in an Anaconda (http://anaconda.org) environment. The second radiologist (with 8 years of CEUS experience) checked the segmentations. If there were any inconsistencies, the results were jointly discussed, and further modifications were made until a consensus was reached. Finally, the patient images and labels were imported into the Darwin Research Platform (https://arxiv.org/abs/2009.00908) for feature extraction and model establishment. The workflow scheme is illustrated in **Figure 1**. The nodule segmentation process is described in the **Supplementary Materials**.

### 2.4 Feature extraction and selection

**Figure 2:**
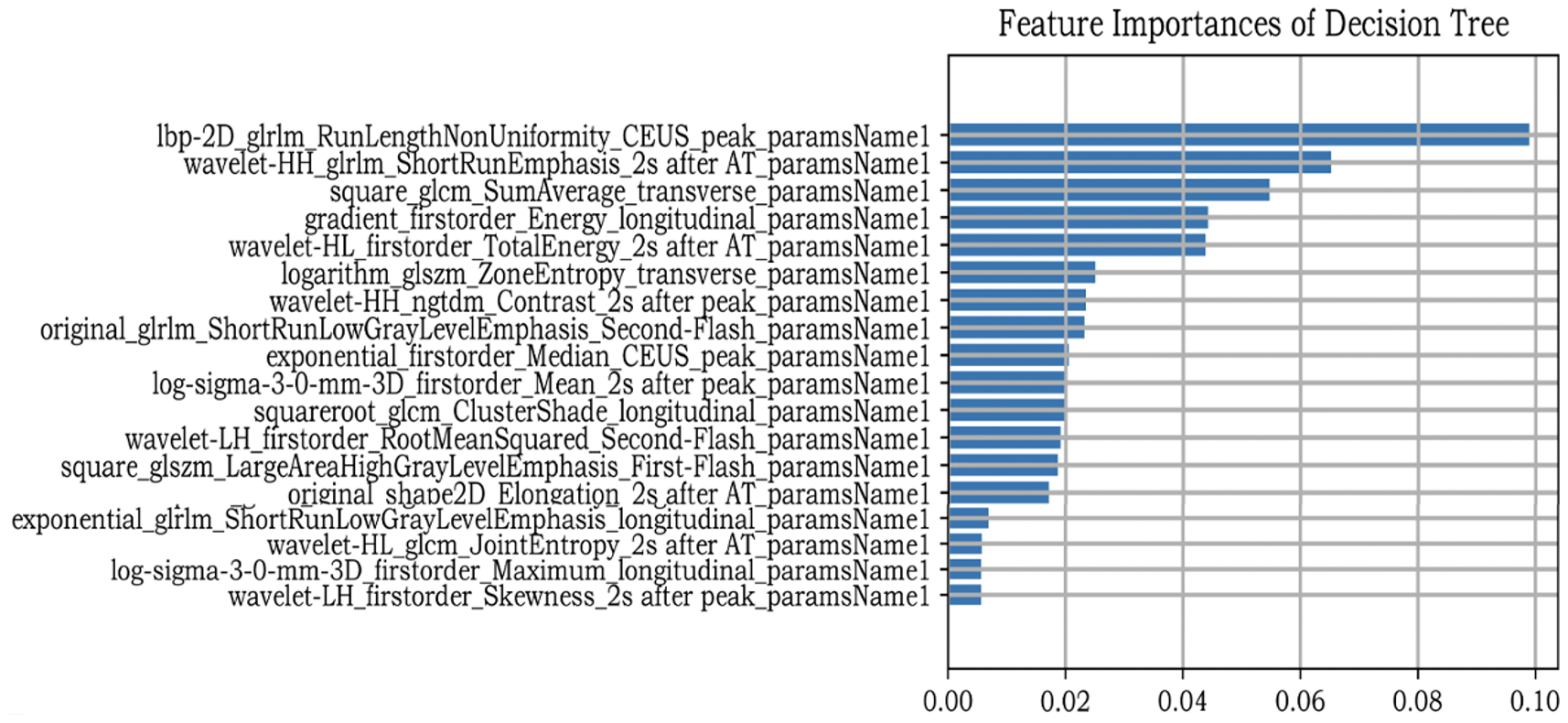
Feature selection.

After nodule segmentation, feature extraction was performed using the “PyRadiomics” package for Python (Python Software Foundation, Beaverton, OR, USA). Radiomics features include first-order, shape, and texture. First-order features can be obtained using a simple metric procedure to clarify the distribution of voxel intensities, such as mean range, variance, and kurtosis. Texture features are used to describe the heterogeneity of the lesion, including the gray-level cooccurrence matrix (GLCM), gray-level run length matrix (GLRLM), gray-level dependence matrix (GLDM), neighboring gray-tone difference matrix (NGTDM), and gray-level size zone matrix (GLSZM). Eight kinds of filters were applied in our study to transform the original images: exponential, gradient, local binary pattern- two dimensional (Lbp-2D), logarithm, square, square root, wavelet, and Laplacian of Gaussian (LoG). First-order shape and texture features were extracted from the derived images. However, since a single image contained 1125 features, seven images from one patient produced 7875 features in total. We extracted all features and subsequently selected them. Feature selection is an important ML procedure because it reduces computational complexity and trains classifiers more accurately. Maximum absolute normalization was used to scale the numerical value to the unit length within a range of –1 to 1. The variance threshold can remove all low-variance features. To reduce overfitting and find definitive correlation features, only F values equal to 0 were excluded from this study. The classifiers also contain algorithms that iteratively calculate the importance of the features. Finally, the decision tree (DT) classifier was used to determine the most relevant feature rankings (**Figure 2**).

### 2.5 Model development

Six ML models, namely support vector machine (SVM), logistic regression (LR), DT, random forest (RF), gradient boosting decision tree (GBDT), and extreme gradient boosting (XGBOOST) were used to determine the best diagnostic performance. The radial basis function was used in the SVM classifier, and the penalty coefficient C was used to set the tolerance for misclassified samples (from 0.0001 to 1,000). LR was based on an elastic net, and the I1 ratio was set to 0.5. For RF, DT, GBDT, and XGBOOST, the maximum depth of the tree was set at 5 to avoid overfitting. If values were missing, we chose the mean value as a supplement. The 10-fold crossvalidation was used to inspect the accuracy of the models. The ROC curve and area under the curve (AUC) were used to compare the performance of the six ML models, and the sensitivity, specificity, accuracy, F1-score, positive predictive value (PPV), and negative predictive value (NPV) were calculated.

### 2.6 Statistical analysis

Statistical analysis was performed using the SPSS software (version 26.0; IBM Corp., Armonk, NY, USA). Count data were recorded as frequencies and rates. The measurement data that confirmed a normal distribution were recorded as mean ± standard deviation, while data that were not consistent with a normal distribution were recorded as the median (interquartile range). Furthermore, measurement data between groups were compared using the independent t-test and Mann–Whitney U test. Count data (clinical data, 2D-US and CEUS data) were analyzed using chi-square or Fisher’s exact tests. Radiomics analyses were performed using Python (version 3.6). Delong’s test was used to test whether there were any differences in AUC among the six ML models and between the ML model and human readers. A calibration curve demonstrated the consistency between the prediction model and the actual situation. Decision curve analysis (DCA) was used to determine whether this model had net clinical benefits. Statistical significance was set at *P <*0.05.

## 3 Results

### 3.1 Clinical and sonographic data

**Figure 3:**
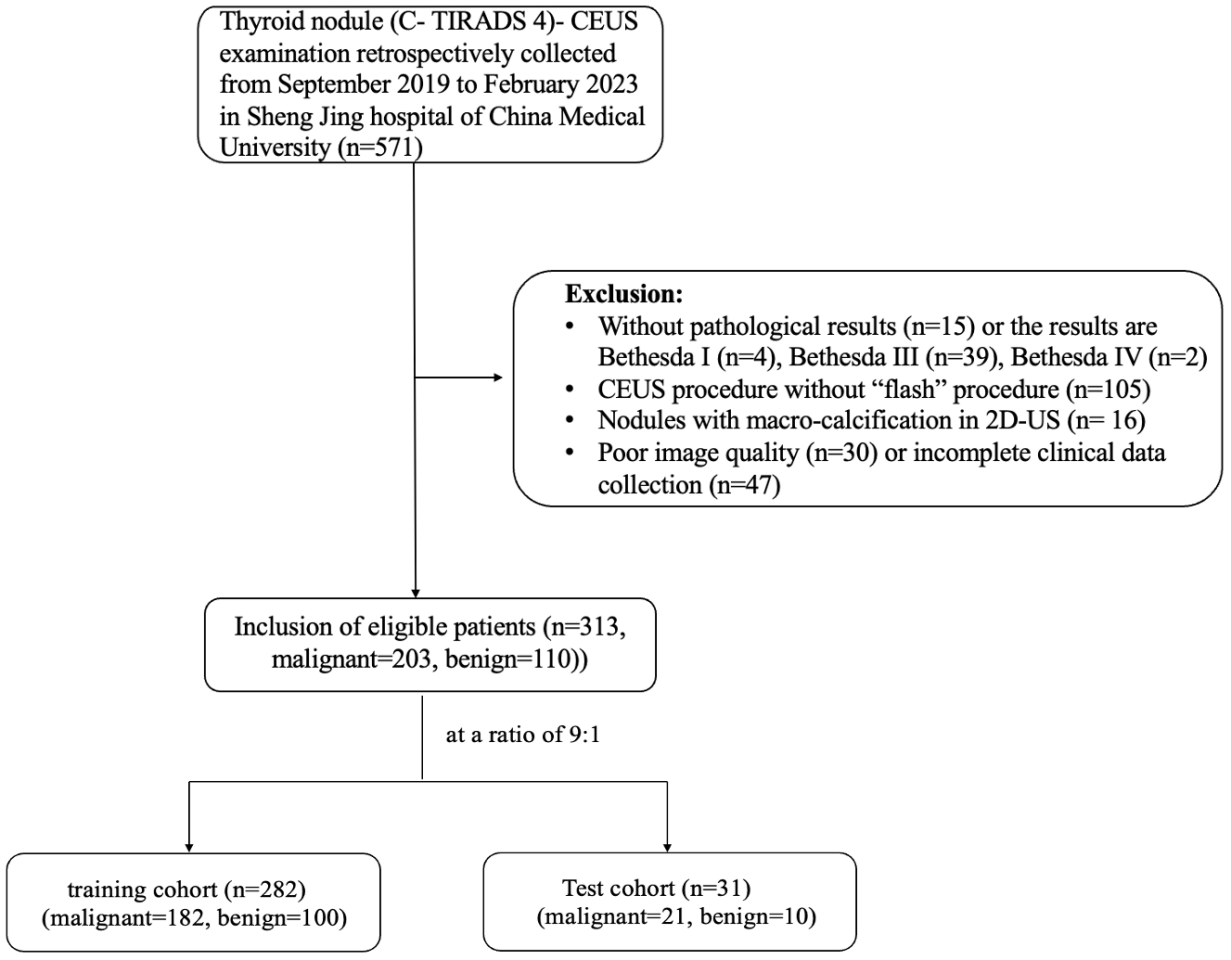
Retrospective workflow. CEUS, contrast-enhanced ultrasound.

A total of 313 nodules were enrolled in our study, with 282 in the training cohort and 31 in the test cohort. The training cohort included 100 benign and 182 malignant nodules, while the test cohort included 10 benign and 21 malignant nodules. In our data, 89 nodules were classified as C- TIRADS 4a, 128 nodules were classified as C- TIRADS 4b, 96 nodules were classified as C- TIRADS 4c. The malignancy rate of C- TIRADS 4a, 4b and 4c were 34.8% (31/89), 70.3% (90/128) and 85.4% (82/96), respectively. The characteristics of the nodules are listed in **Table 1**, and the patient inclusion flowchart is shown in **Figure 3**. In the training cohort, the clinical and sonographic variables between the malignant and benign groups showed significant differences in age, number, size, solid composition, microcalcification, shape, margin, enhanced intensity, homogeneity, and wash-in patterns (all *P*<0.05). However, no significant difference was found in sex, location, Hashimoto’s background, echogenicity, centripetal enhancement, and wash-out patterns (all *P >*0.05). There was no statistically significant difference in the distribution of patients between the training and test cohorts (*P >*0.05).

**Table 1:** Clinical and sonographic characteristics.

	Training cohort (*n*=282)				Test cohort (*n =* 31)	*P*
Characteristics	Total (*n* = 282)	Benign(*n* = 100)	Malignant (*n* = 182)	*p*		
Age (years)	44.57 ± 12.31	48.12 ± 12.5	42.62 ± 11.79	0.000*	45.52 ± 11.47	0.683
Sex Female Male	224 (79.4%) 58 (20.6%)	82 (82.0%) 18 (18.0%)	142 (78.0%) 40 (22.0%)	0.429	23 (74.2%) 8 (25.8%)	0.497
Number Single Multiple	99 (35.1%) 183 (64.9%)	19 (19.0%) 81 (81.0%)	80 (44.0%) 102 (56.0%)	0.000*	12 (38.7%) 19 (61.3%)	0.691
Size (mm) Maximum diameter	10.67 ± 9.07	15.1 ± 12.14	8.24 ± 5.52	0.000*	10.2 ± 7.83	0.779
Location Upper pole Middle Subthyroid pole Isthmus	64 (22.7%) 112 (39.7%) 77 (27.3%) 29 (10.3%)	23 (23.0%) 37 (37.0%) 34 (34.0%) 6 (6.0%)	41 (22.5%) 75 (41.3%) 43 (23.6%) 23 (12.6%)	0.133	10 (32.3%) 10 (32.2%) 7 (22.6%) 4 (12.9%)	0.595
Hashimoto Background Yes No	46 (16.3%) 236 (83.7%)	17 (17.0%) 83 (83.0%)	29 (15.9%) 153 (84.1%)	0.917	7 (22.6%) 24 (77.4%)	0.377
Solid composition Yes No	278 (98.6%) 4 (1.4%)	96 (96.0%) 4 (4.0%)	182 (100%) 0	0.007*	31 (100%) 0	1.000
Very low echogenicity Yes No	16 (5.7%) 266 (94.3%)	3 (3.0%) 97 (97.0%)	13 (7.1%)) 169 (92.9%)	0.150	2 (6.5%) 29 (93.5%)	0.860
Microcalcification Yes No	89 (31.6%) 193 (68.4%)	24 (24.0%) 76 (76.0%)	65 (35.7%) 117 (64.3%)	0.043*	5 (16.1%) 26 (83.9%)	0.075
Shape (Aspect ratio) >1 <1	81 (28.7%) 201 (71.3%)	12 (12.0%) 88 (88.0%)	69 (37.9%) 113 (62.1%)	0.000*	9 (29.0%) 22 (71.0%)	0.971
Margin Regular Irregular	144 (51.1%) 138 (48.9%)	72 (72.0%) 28 (28.0%)	72 (39.6%) 110 (60.4%)	0.000*	19 (61.3%) 12 (38.7%)	0.279
Enhanced intensity Hyperenhancement Iso-enhancement Hypoenhancement	54 (19.2%) 149 (52.8%) 79 (28.0%)	36 (36.0%) 32 (32.0%) 32 (32.0%)	18 (9.9%) 47 (25.8%) 117 (64.3%)	0.000*	10 (32.3%) 16 (51.6%) 5 (16.1%)	0.148
Homogeneity Homogeneous Heterogeneous	108 (38.3%) 174 (61.7%)	56 (56.0%) 44 (44.0%)	52 (28.6%) 130 (71.4%)	0.000*	16 (51.6%) 15 (48.4%)	0.150
Centripetal enhancement Yes No	28 (9.9%) 254 (90.1%)	9 (9.0%) 91 (91.0%)	19 (10.4%) 163 (89.6%)	0.699	3 (10%) 28 (90%)	1.000
Wash-in Synchronous Later Earlier	133 (47.2%) 116 (41.1%) 33 (11.7%)	51 (51.0%) 29 (29.0%) 20 (20.0%)	82 (45.1%) 87 (47.8%) 13 (7.1%)	0.001*	20 (64.5%) 9 (29.0%) 2 (6.5%)	0.180
Wash-out Synchronous Later Earlier	180 (63.8%) 44 (15.6%) 58 (20.6%)	64 (64.0%) 12 (12.0%) 24 (24.0%)	116 (63.7%) 32 (17.6%) 34 (18.7%)	0.337	22 (71.0%) 4 (12.9%) 5 (16.1%)	0.731

*Represents P <0.05. Numerical data are presented as mean ± standard deviation. Categorical data are presented as numbers (%).

### 3.2 The US and CEUS analysis by human reader

**Figure 4:**
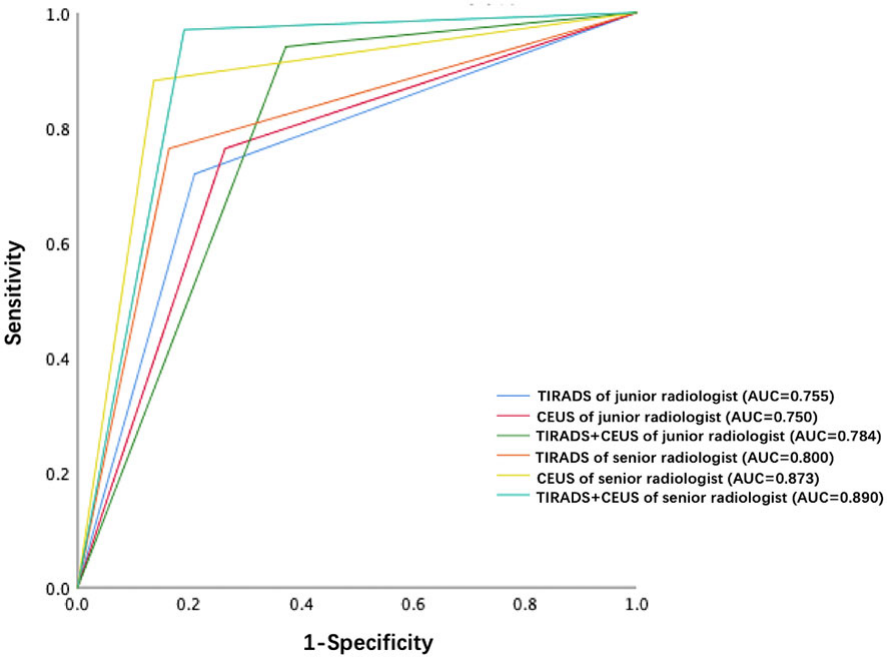
ROC curves of TIRADS, CEUS and TIRADS combined with CEUS of junior radiologist and senior radiologist, respectively.

Each nodule was evaluated simultaneously by a junior radiologist (3 years of CEUS experience) and a senior radiologist (8 years of CEUS experience). The parallel method was used for combined diagnosis of C- TIRADS and CEUS. That is to say, if both C-TIRADS and CEUS were benign, the final diagnosis was recorded as benign, while one of the C-TIRADS or CEUS was malignant, the final diagnosis was recorded as malignant. As shown in **Table 2**; **Figure 4**, the AUCs of junior radiologist observing US for C-TIRADS classification, CEUS videos, and the combined diagnosis of the two methods were 0.755, 0.750, 0.784, respectively. Except from the specificity and the PPV, the sensitivity, NPV and accuracy of combining US and CEUS by junior radiologist were higher than using US and CEUS alone, which were 0.941, 0,852, 0.831, respectively. The AUC of senior radiologist observing US for C-TIRADS classification, CEUS video, and combined diagnosis of the two methods were 0.80, 0.873 and 0.890 respectively. Except from the specificity and the PPV, the sensitivity, NPV and accuracy of combining US and CEUS by senior radiologist were higher than using US and CEUS alone, which were 0.970, 0,937, 0.914, respectively.

**Table 2:** The US and CEUS analysis by human readers.

Models	SEN	SPE	PPV	NPV	Accuracy	AUC
Junior radiologist C- TIRADS	0.720 (0.651, 0.779)	0.791 (0.701, 0.860)	0.864 (0.801, 0.910)	0.604 (0.519, 0.683)	0.744	0.755 (0.698, 0.812)
Junior radiologist CEUS	0.764 (0.698, 0.819)	0.736 (0.642, 0.814)	0.842 (0.780, 0.890)	0.628 (0.538, 0.710)	0.754	0.750 (0.692, 0.808)
Junior radiologist C- TIRADS+CEUS	0.941 (0.897, 0.968)	0.627 (0.529, 0.716)	0.823 (0.767, 0.869)	0.852 (0.752, 0.918)	0.831	0.784 (0.698, 0.812)
Senior radiologist C- TIRADS	0.768 (0.703, 0.823)	0.836 (0.751, 0.898)	0.897 (0.839,0.936)	0.662 (0.576, 0.739)	0.792	0.800 (0.747, 0.853)
Senior radiologist CEUS	0.882 (0.827, 0.921)	0.864 (0.782, 0.920)	0.923 (0.873, 0.955)	0.800 (0.713, 0.864)	0.875	0.873 (0.828, 0.918)
Senior radiologist C- TIRADS+CEUS	0.970 (0.934, 0.988)	0.809 (0.721, 0.875)	0.904 (0.855, 0.938)	0.937 (0.862, 0.974)	0.914	0.890 (0.844, 0.936)

C- TIRADS, Chinese version of thyroid imaging reporting and data system; CEUS, contrast-enhanced ultrasound; PPV, positive predictive value; NPV, negative predictive value; AUC, area under the receiver operating characteristic curve; SEN, sensitivity; SPE, specificity; PPV, positive predictive value; NPV, negative predictive value.

### 3.3 Prediction performance of ML models based on 2D-US combined with CEUS key frames

**Figure 5:**
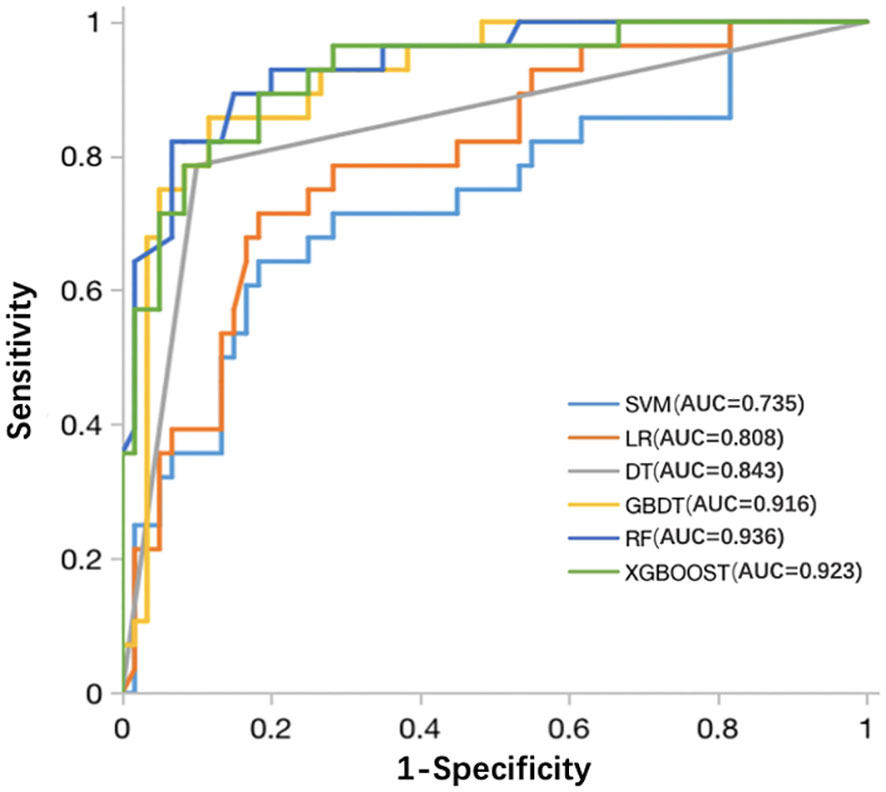
ROC curves of the SVM, LR, DT, RF, GBDT, and XGBOOST classifiers in the test cohort. ROC, receiver operating characteristic; SVM, support vector machine; LR, logistic regression; DT, decision tree; RF, random forest; GBDT, gradient boosting decision tree; XGBOOST, extreme gradient boosting.

**Figure 6:**
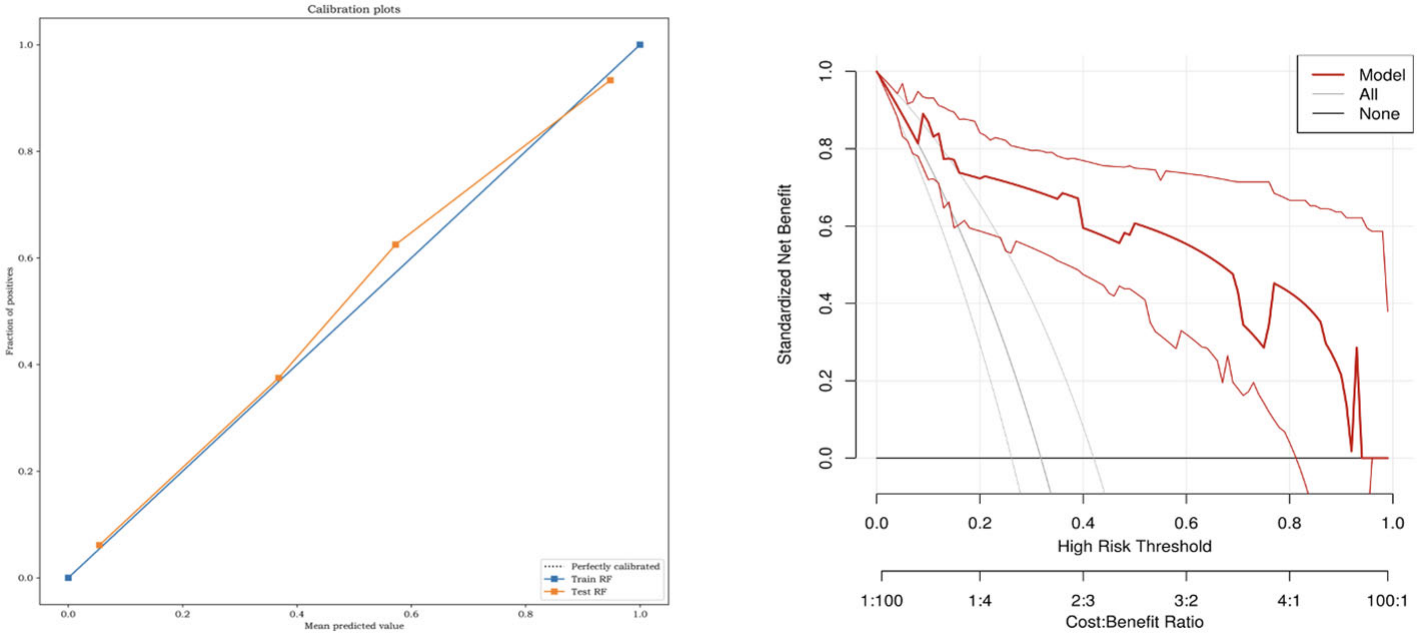
The calibration curves and decision curve analysis of RF models. RF, random forest.

**Figure 7:**
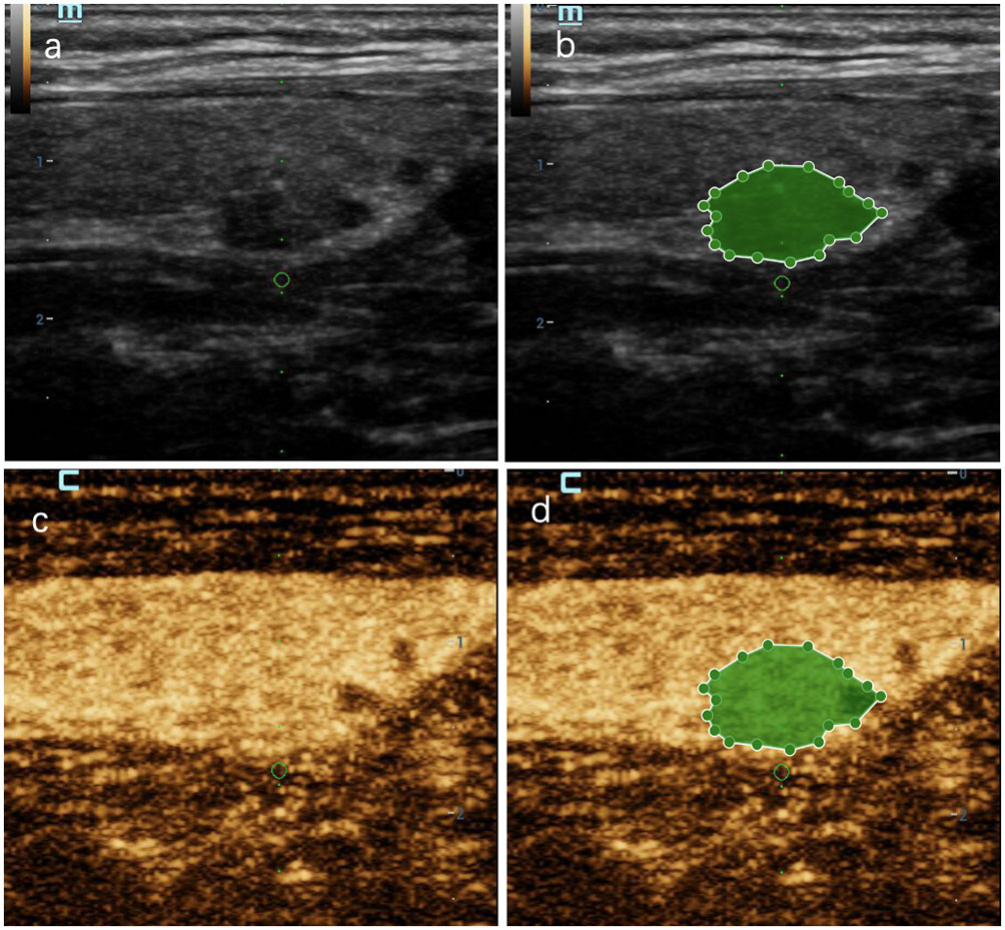
A thyroid nodule in left lobe in a 46-year-old woman in test cohort. **(A)** 2D-US image; **(B)** the mask image corresponding to 2D-US image; **(C)** CEUS image at peak time; **(D)** the mask image of CEUS image at peak time. The nodule was solid, hypoechoic, blurred margin, aspect ratio less than 1, with microcalcification and was categorized as C-TIRADS 4c. CEUS showed “later wash-in, heterogeneous enhancement” and “later wash-out”, and was diagnosed as malignant. RF model classifies it as malignant. Histologic analysis revealed papillary microcarcinoma (PTMC).

**Figure 8:**
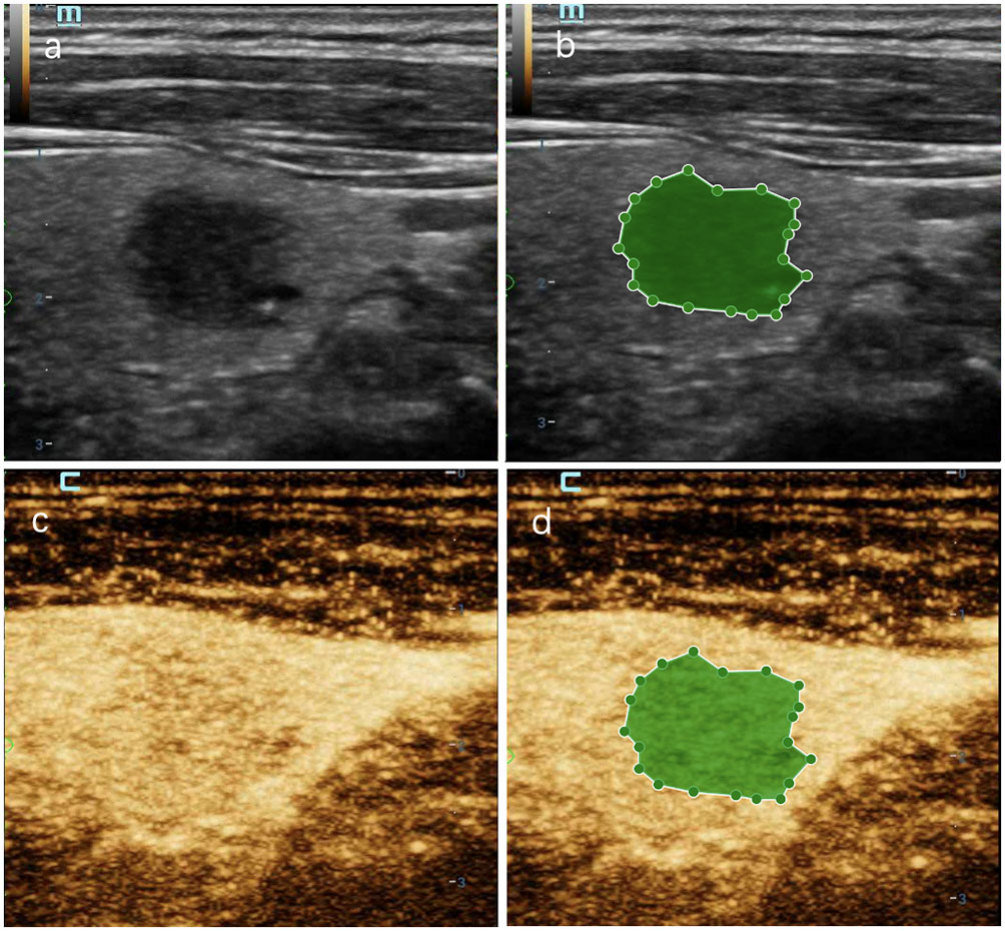
A thyroid nodule in right lobe in a 56-year-old man in test cohort. **(A)** 2D-US image; **(B)** the mask image corresponding to 2D-US image; **(C)** CEUS image at peak time; **(D)** the mask image of CEUS image at peak time. The nodule was solid with blurred margin and was categorized as C-TIRADS 4b. CEUS showed “later wash-in” and “with hypointensity”, and was diagnosed as malignant. RF model classifies it as benign. Histologic analysis revealed nodular goiter with granuloma formation.

The six classifiers (SVM, LR, DT, RF, GBDT, and XGBOOST) and their performance are listed in **Table 3**. AUCs for SVM, LR, DT, RF, GBDT, and XGBOOST in the training cohort were 0.75, 0.87, 1.00, 1.00, 1.00, and 0.92, respectively. In the test cohort, AUCs of SVM, LR, DT, RF, GBDT, and XGBOOST were 0.74, 0.81, 0.84, 0.94, 0.92, and 0.92, respectively. The ROC curves of the six ML models are shown in **Figure 5**. The results of the Delong test showed that in the test cohort, the difference between AUC of SVM, LR, and DT was not statistically significant (*P >*0.05). Similarly, the difference in AUC between RF, XGBOOST, and GBDT was not statistically significant (*P >*0.05). RF, GBDT, and XGBOOST had comparable predictive effectiveness. The differences in AUC between GBDT, LR, and DT were not statistically significant (*P >*0.05); however, AUCs of RF and XGBOOST were statistically significant compared to those of SVM, LR, and DT, respectively (all *P <*0.05). Notably, AUC of RF was the highest in the test cohort (0.94). Additionally, the calibration and DCA curves of RF showed favorable consistency with reality (**Figure 6**). The cases in test cohorts were presented in **Figures 7**, **8**.

**Table 3:** Predictive performance of six machine learning models based on 2D-US and CEUS key frames.

Parameter	SVM		LR		DT		RF		GBDT		XGBOOST	
	Training cohort	Test cohort	Training cohort	Test cohort	Training cohort	Test cohort	Training cohort	Test cohort	Training cohort	Test cohort	Training cohort	Test cohort
AUC	0.746 (0.707–0.786)	0.735 (0.615–0.854)	0.867 (0.839–0.895)	0.808 (0.709– 0.907)	1	0.843 (0.757–0.929)	1	0.936 (0.884–0.988)	0.999 (0.998–1)	0.916 (0.854–0.978)	1	0.923 (0.864–0.984)
ACC	0.741	0.75	0.791	0.807	1	0.864	1	0.898	0.99	0.864	1	0.841
SEN	0.671 (0.61–0.726)	0.643 (0.458–0.793)	0.813 (0.761–0.857)	0.679 (0.493, 0.821)	1 (0.985–1)	0.786 (0.605–0.898)	1 (0.985,1)	0.821 (0.644–0.921)	0.988 (0.966–0.996)	0.857 (0.685–0.943)	1 (0.985–1)	0.893 (0.728–0.963)
SPE	0.773 (0.736–0.807)	0.8 (0.682–0.882)	0.781 (0.744–0.814)	0.867 (0.758, 0.931)	1 (0.993–1)	0.9 (0,799–0.953)	1 (0.993,1)	0.933 (0.841–0.974)	0.991 (0.978–0.996)	0.867 (0.758–0.931)	1 (0.993–1)	0.817 (0.701–0.894)
PPV	0.581 (0.523–0.636)	0.6 (0.423–0.754)	0.635 (0.581–0.685)	0.704 (0.515–0.841)	1 (0.985–1)	0.786 (0.605–0.898)	1 (0.985–1)	0.852 (0.675–0.941)	0.98 (0.955–0.992)	0.75 (0.579–0.867)	1 (0.985–1)	0.694 (0.531–0.82)
NPV	0.834 (0.798–0.864)	0.828 (0.711–0.904)	0.899 (0.869–0.923)	0.852 (0.743–0.92)	1 (0.993–1)	0.9 (0.799–0.953)	1 (0.993–1)	0.918 (0.822–0.964)	0.994 (0.984–0.998)	0.929 (0.83–0.972)	1 (0.993–1)	0.942 (0.8440.98)
F1-Score	0.741	0.621	0.713	0.691	1	0.786	1	0.836	0.984	0.8	1	0.781

SVM, support vector machine; LR, logistic regression; DT, decision tree; RF, random forest; GBDT, gradient boosting decision tree; XGBOOST, extreme gradient boosting; AUC, area under curve; ACC, accuracy; SEN, sensitivity; SPE, specificity; PPV, positive predictive value; NPV, negative predictive value.

### 3.4 Comparison with human readers

**Figure 9:**
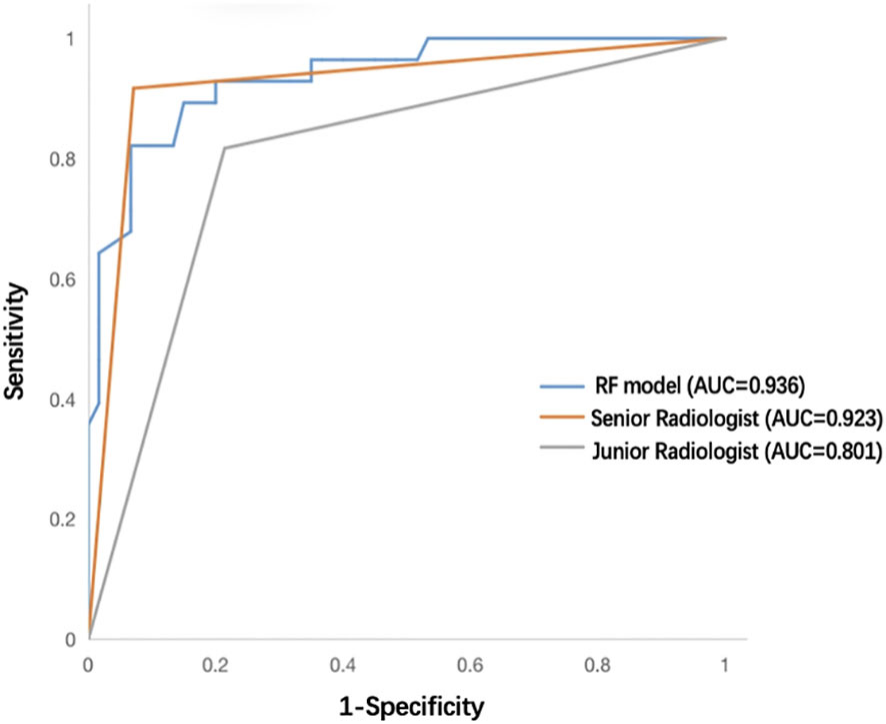
ROC curves of the RF model, senior radiologist, and junior radiologist in the test cohort. ROC, receiver operating characteristic; RF, random forest.

A senior radiologist (8 years of CEUS experience) and a junior radiologist (3 years of CEUS experience) independently reviewed the transverse and longitudinal sections of the test cohort’s 2D-US and CEUS videos of each nodule. Both groups were blinded to clinical characteristics and pathological results, and a definitive diagnosis of whether each nodule was benign or malignant was provided. The diagnostic performances of the best-performing RF model and human readers are summarized in **Table 4**; **Figure 9**. As shown, the RF model achieved an equivalent performance to that of the senior radiologist (*P* = 0.799) and gained more specificity. The RF model outperformed the junior radiologist (*P* = 0.039) and showed greater sensitivity, specificity and NPV.

**Table 4:** Diagnostic performance of the RF model compared to human readers in the test cohort.

	Models	SEN	SPE	PPV	NPV	Accuracy	AUC	*P*
Test cohort	RF model	0.821 (0.644–0.921)	0.933 (0.841–0.974)	0.852 (0.675–0.941)	0.918 (0.822–0.964)	0.898	0.936 (0.884–0.988)	
	Senior radiologist	0.965 (0.868–0.994)	0.839 (0.655–0.939)	0.917 (0.808–0.968)	0.929 (0.750–0.988)	0.920	0.923 (0.854–0.991)	0.799
	Junior radiologist	0.817 (0.691–0.901)	0.786 (0.585–0.910)	0.891 (0.771–0.955)	0.667 (0.481–0.814)	0.807	0.801 (0.696–0.906)	0.039*

RF, random forest; SEN, sensitivity; SPE, specificity; PPV, positive predictive value; NPV, negative predictive value; AUC, area under the curve.

## 4 Discussion

In this study, we constructed six ML models using 2D-US images combined with five CEUS keyframes. The ROC curves showed that the diagnostic performance of our models was desirable, with all AUC values >0.80 in the test cohort (except SVM [0.74]). Moreover, we compared our best model with human readers (senior and junior radiologists) and found that the best ML model achieved equivalent performance to that of the senior radiologist and outperformed the junior radiologist.

Traditional diagnostic methods for thyroid nodules, such as 2D-US, color Doppler flow imaging (CDFI), elastography, and FNA, have many disadvantages (35–37); the main ones are severe overdiagnosis and overtreatment (38, 39). For example, some Hashimoto’s nodules may show hypoechogenicity with blurred margins on 2D-US, which may be classified as TIRADS >4 and require unnecessary FNA according to the guidelines (7, 40, 41). CEUS, as a novel noninvasive microangiography technology, can reveal microvasculature with a smaller diameter (>40 µm) than that by CDFI (>100 µm) and is helpful in the detection of malignant thyroid nodules (42, 43). Recent studies have indicated that CEUS could modify the current TIRADS to create a new risk stratification that may reduce unnecessary biopsies (42–46). Our team had published one CEUS- TIRADS model to differentiate thyroid nodules (C-TIRADS 4) by combining CEUS with C-TIRADS (46), which had high clinical practicability in clinic. Additionally, CEUS images may contain valuable information that has not received sufficient attention in daily clinical practice. In recent years, AI, especially radiomic features, has demonstrated promising potential for evaluating the characteristics of thyroid nodules (47, 48). Radiomics has also been used to diagnose cytologically uncertain nodules (49–51), lymph node metastases (52, 53), and extrathyroidal extension (54). Many studies employing AI for evaluating the thyroid are mainly based on 2D-US images (48, 55, 56). In 2015, LeCun introduced the principles of deep learning and convolutional neural networks (CNNs) (18), attracting the interest of many researchers. The principle of machine or deep learning is that CNNs are trained using a large number of 2D-US images with known corresponding pathological results. A specific algorithm is used to segment US images. After several calculation iterations, the CNNs can capture and analyze thyroid nodules and suggest risk stratification. Studies on ML based on 2D-US to distinguish malignant thyroid nodules from benign nodules could reach a diagnostic accuracy of approximately 90%. Peng et al. developed a deep learning AI model based on 2D-US to diagnose thyroid nodules that outperformed 12 radiologists (AUC: 0.922 vs. 0.839, *P <*0.05) (37). Conversely, a study conducted by Sun et al., also based on 2D-US, indicated that the experts achieved better performance (AUC: 0.881 vs. 0.819) (57). Gong et al. reported that an AI-assisted diagnostic system combined with CEUS could significantly improve the diagnostic sensitivity and NPV in diagnosing thyroid nodules classified as American College of Radiology Thyroid Imaging (ACR-TIRADS) 4 (58). However, to our knowledge, few researchers have developed AI models based on CEUS images. To date, only two studies have proposed AI diagnostic models based on CEUS image information (27, 28). Wan et al. used DL to build a diagnostic model based on dynamic CEUS video and obtained AUC of 0.92 (27), which was lower than ours (AUC: 0.94); ACC in their study was substantially lower than that in ours (0.80 vs. 0.90). Guo et al. used logistic regression to build ML models based on US and CEUS features, while as for CEUS features, only a single frame of CEUS images was used (28). Our studies extracted radiomics features five key CEUS frames and the sample size of our study is bigger (313 vs. 123). And our study aimed at thyroid nodules which are classified as C-TIRADS 4, which are relatively hardly differentiated in clinic. Therefore, this was the first study to provide the highest value of radiomics information from CEUS images in thyroid nodules (C-TIRADS 4) evaluation, offering a promising, noninvasive, fast, feasible, and reliable method.

In our study, none of the patients experienced complications during CEUS and FNA. By comparing the FNA and surgical pathological results from January 2016 to June 2021 in our hospital, we found that the success rate and diagnostic accuracy of FNA were 96.6% and 93.3%, respectively (59). The accuracy of FNA was much higher than that in most previous studies, indicating that the pathological results from FNA at our institution were reliable. Our study also demonstrated that malignant thyroid nodules commonly occurred in younger people (*P <*0.05). The statistical differences between malignant and benign nodules in the training cohort were also significant for nodule number, nodule size, nodule composition, the presence of microcalcifications, shape, margin, enhanced intensity of CEUS, homogeneity of CEUS, and wash-in patterns of CEUS (all *P <*0.05). Regarding CEUS patterns, the malignant nodules in our data mostly showed hypoenhancement (117/182; 64.3%), heterogeneous enhancement (130/182; 71.4%), and later wash-in (87/182; 47.8%), which is consistent with previous studies (12, 33, 34). This may be attributed to the peripheral blood vessels of malignant nodules being damaged by malignant growth, hindering contrast agent entry. When the nodule is small, the number of new blood vessels, branches, and arteriovenous fistulas is not relatively large, and the inside of the nodule will be closely related to the poor blood supply and uneven distribution of blood vessels within the malignant nodules. In the present study, the mean maximal diameter of malignant nodules was smaller than that of benign nodules (*P <*0.05), which may indicate that the direction of perfusion of contrast agents was difficult to observe, which explains the lack of statistical significance in the enhancement methods and wash-out patterns. And in our data, the diagnostic AUC and accuracy of both junior and senior radiologist of using US combined with CEUS were higher than those of US or CEUS alone.

In this study, we first extracted nearly all radiomics features as published in the present literature. Subsequently, we adopted maximum abs normalization to preprocess the data. Many data normalization methods are used in ML, such as Z-score standardization, max abs normalization, min-max normalization, robust scaling, and median absolute deviation. The advantage of max absol normalization lies in its ability to retain data distribution without centralizing it, preserving the sparsity of large-scale data such as ours. We then used the variance threshold to eliminate outliers from the data. DT is a nonparametric method. Thus, it does not make any assumptions regarding the spatial distribution or categorical structure of the data, making it suitable for our study. The best feature selection is based on the DT classifier. Wavelet features accounted for the largest proportion of radiomics features (6/18). High-dimensional wavelet features are texture features that show lesion heterogeneity (60). Fan et al. used ML to predict the aggressiveness of prostate cancer, and wavelet features accounted for the largest proportion of their models (61). Meng et al. and Aerts et al. reached similar conclusions (60, 62). Additionally, CEUS frames played a substantial role in feature selection (12/18), illustrating the importance of CEUS images. Moreover, among the selected features, the top-ranked one was the “time to peak” frame. This may be because the image is brightest at the peak time, and the number of microbubbles in the nodule area is the highest, which can probably provide more information.

Classifiers play a crucial role in ML procedures. Our study uses six classifiers for model development(SVM, LR, DT, RF, GBDT, and XGBOOST). Support vector machine (SVM) is a kind of generalized linear classifier for binary classification of data according to supervised learning, which is more suitable for dealing with complex nonlinear equations than logistic regression. Compared with SVM, LR can be used for multivariate classification and is more suitable for small data volume. Decision tree (DT) is a basic classification and regression method and defined as a conditional probability distribution on feature space and class space. Both random forest (RF) and gradient boosting decision tree (GBDT) are based on DT. RF is an extension of a parallel ensemble learning method, and “random” means the randomness of the selected partition attributes. GBDT is a decision tree model trained with gradient boosting strategy, which performs well in screening features (63). XGBOOST is a kind of basic GBDT, but compared with GBDT, it can support custom loss functions and add more regular terms, handling of missing value and column sampling. Among the four models based on DT, RF can converge to a lower generalization error than the traditional DT. What is more, DT selects the optimal partition attribute from all attribute sets, while RF selects the partition attribute only in a subset of the attribute set, so the training efficiency is higher. And each tree of RF only chooses part of samples and features, breaking through the “overfitting” defect of DT. Compared with GBDT and XGBOOST, the performance of RF is more stable, the parameter adjusting is relatively less complicated, the operation time is short, and the universality is stronger. Compared with SVM and LR, RF randomly selects samples and features for each tree, removes noise variables, increases noise resistance and provides more stable performance. Moreover, unlike SVM, as the number of observed samples and features increases, SVM firstly needs to spend much time to find a suitable kernel function during the calculation. RF has no such weakness. The results of our study also proved that RF was the optimal classifier for our model. In our data, the RF, GBDT, and XGBOOST classifiers generally performed better than the SVM, LR, and DT classifiers. The RF model performed the best (AUC: 0.94, 95% CI: 0.884–0.988; ACC: 0.90). In the test cohort, our RF model obtained an equivalent performance to that of the senior radiologist (AUC: 0.94 vs.0.92, P = 0.798; ACC: 0.90 vs. 0.92) and was considerably higher in specificity than both the senior (0.93 vs. 0.84) and junior (0.93 vs. 0.79) radiologists. The good performance of our model also indicated that during the CEUS process, the radiologists could pay more attention to those five time points: “2nd second after the arrival time,” “time to peak” frame, “2nd second after peak” frame, “first-flash” frame, and “second-flash” frame, especially the peak time. This not only achieves comparable performance in diagnosing thyroid nodules, which are classified as C- TIRADS 4, but also saves radiologists time compared to watching the entire CEUS video.

This study had some limitations. First, this was a single-center retrospective study; our institution is a referral center, and the malignancy risk of thyroid nodules is relatively high, which may have led to selection bias in our samples. Second, this study lacked external verification, requiring a multi-center, multi-hospital, multi-region study to augment the robustness and generalizability of our results. Third, the ROI lines of the nodules were all manually delineated, and key-frame selection was also observed and operated by radiologists, although we had obtained rather good performance; however, these two procedures are time-consuming and prone to errors, and their efficiency and accuracy could potentially be improved with the implementation of a mature automated artificially intelligent system.

## 5 Conclusion

Our study established six ML models based on two 2D-US images and five CEUS key frames to distinguish malignant from benign thyroid nodules which were classified as C-TIRADS 4. Our study highlighted the information of CEUS image extracted by ML that could not be seen by human eyes, indicating that CEUS may have great potential in the field of thyroid nodules. The RF model, as the optimal ML algorithm, may provide a noninvasive, convenient, feasible, and highly accurate method for invasive FNA and assist junior radiologists in diagnosis or preoperative prediction models. Further studies will address these limitations, making it possible to improve clinical diagnostic and therapeutic strategies.

## Data availability statement

The original contributions presented in the study are included in the article/**Supplementary Material**. Further inquiries can be directed to the corresponding author.

## Ethics statement

The studies involving humans were approved by Medical Ethics Committee of Shengjing Hospital, China Medical University. The studies were conducted in accordance with the local legislation and institutional requirements. The participants provided their written informed consent to participate in this study.

## Author contributions

J-hC: Conceptualization, Data curation, Formal analysis, Investigation, Methodology, Project administration, Software, Supervision, Validation, Visualization, Writing – original draft, Writing – review & editing. Y-QZ: Conceptualization, Methodology, Software, Validation, Visualization, Writing – review & editing. T-tZ: Conceptualization, Data curation, Formal analysis, Methodology, Resources, Writing – review & editing. QZ: Conceptualization, Formal analysis, Investigation, Methodology, Validation, Visualization, Writing – review & editing. A-xZ: Data curation, Formal analysis, Writing – review & editing. YH: Conceptualization, Data curation, Funding acquisition, Methodology, Resources, Supervision, Writing – review & editing.
