# Supplementary material for: Applying machine-learning models to differentiate benign and malignant thyroid nodules classified as C-TIRADS 4 based on 2D-ultrasound combined with five contrast-enhanced ultrasound key frames
Source: Front Endocrinol (Lausanne). 2024 Apr 3;15:1299686. doi: 10.3389/fendo.2024.1299686 (PMC11021584; doi:10.3389/fendo.2024.1299686)
Supplement: Supplementary file 1 [file DataSheet_1.docx]

Supplementary Material- The details of nodule segmentation

Among all the frames cut out from CEUS videos in two devices were displayed simultaneously with corresponding US frames in one view (Fig.S1). In order to delineate the margin of the nodule more precisely, the boundary of the ROI was manually delineated in US images, following which the open-source software Labelme could generate “target” file where the mask was saved. We used the screenshot tool to crop the CEUS frames and the corresponding mask with strict coordinate system. The size of frames from iu22 and Resona9 are 360*380 and 395*495 respectively. In principle, the ROI line should include the entire margin of the nodule ( Fig.S2).


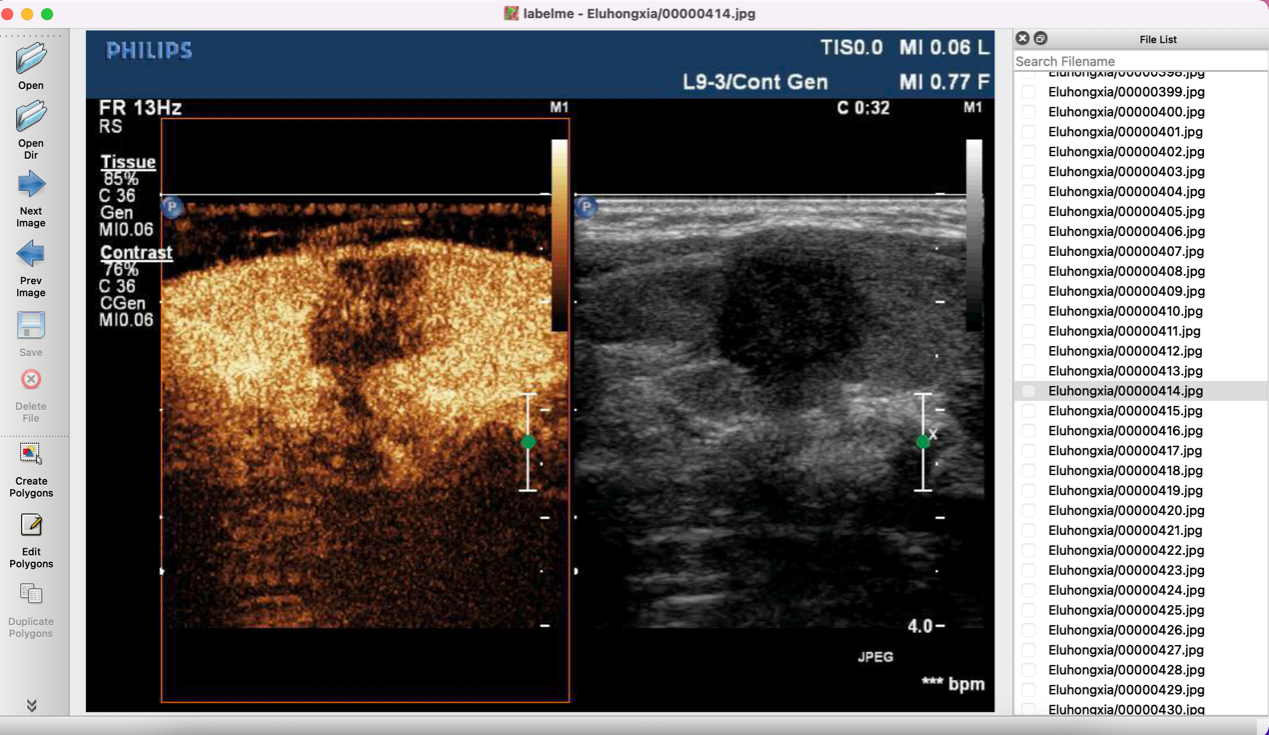


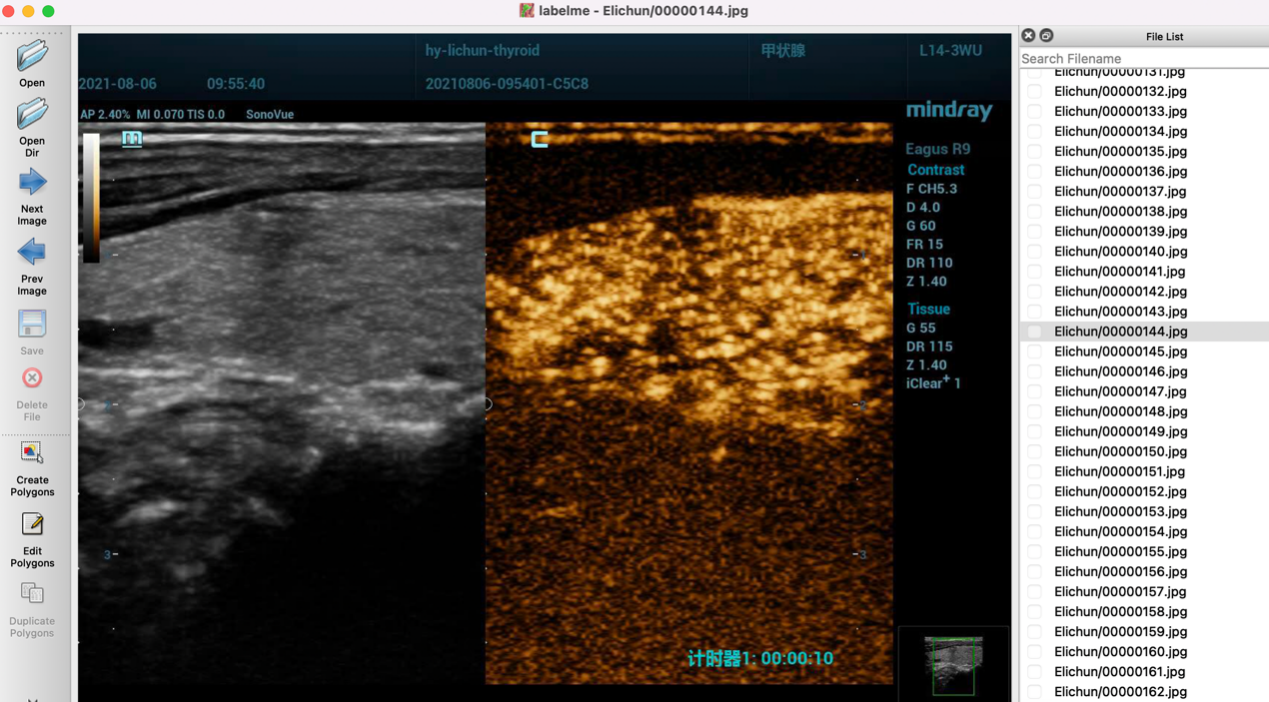


**Supplementary Figure 1.** The CEUS frames cut out from CEUS videos were displayed simultaneously with corresponding US frames in iu22 device and Resona9 device respectively.


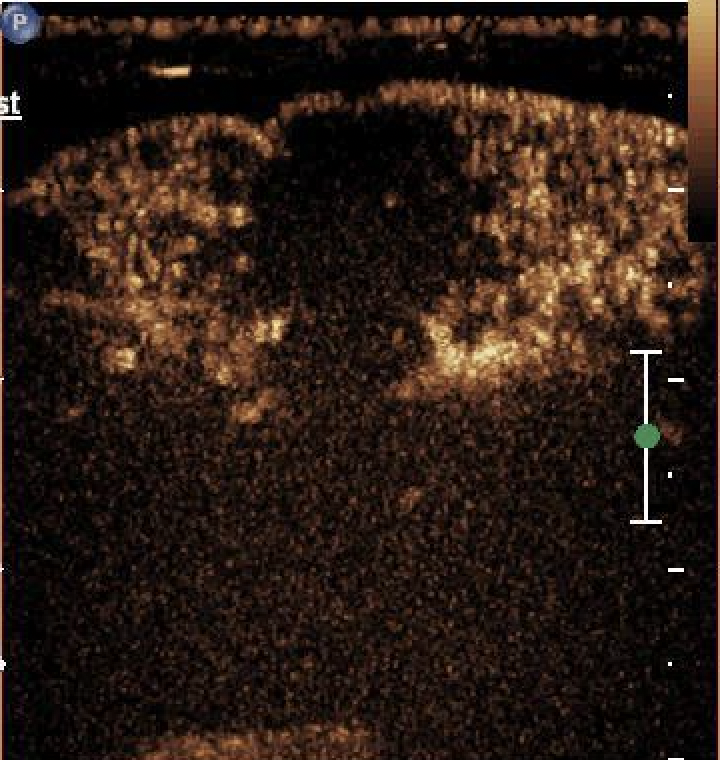


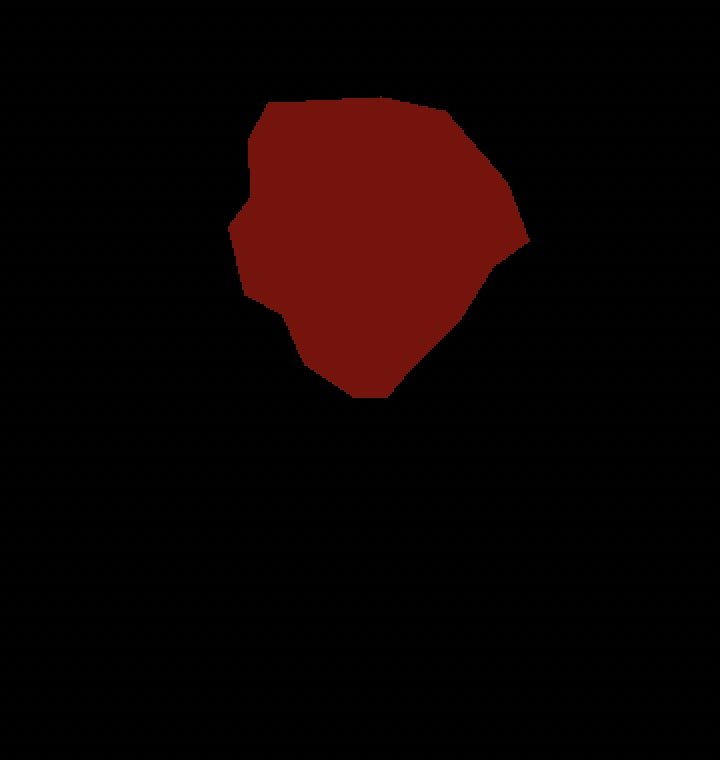


**
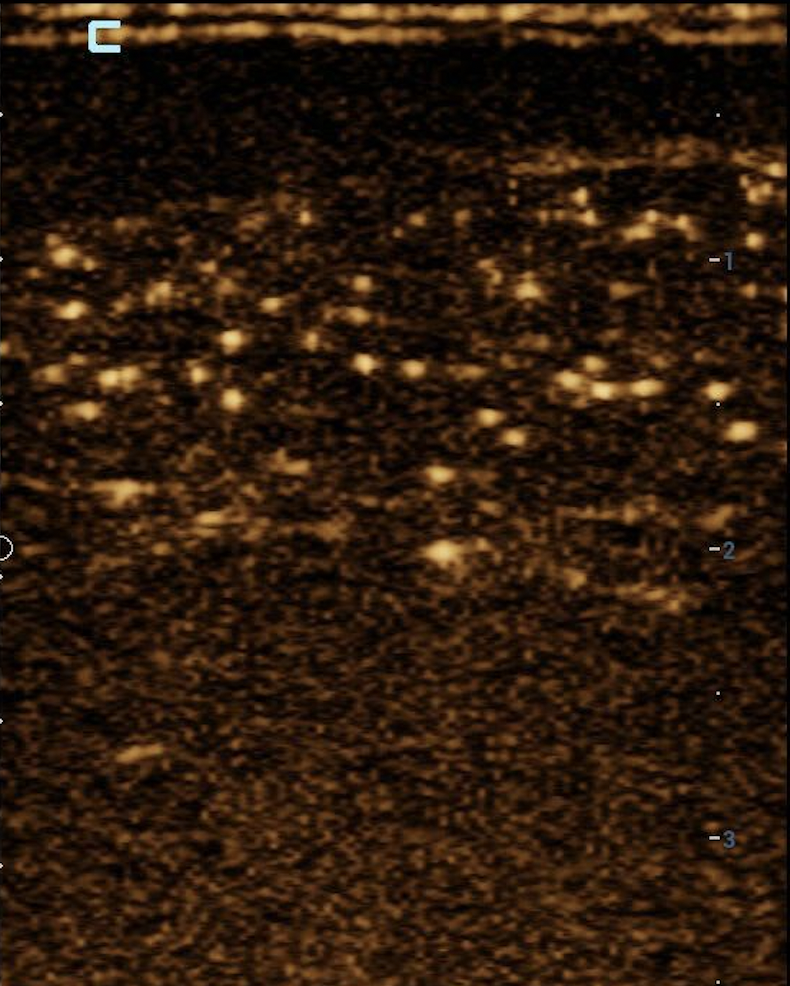
**

**
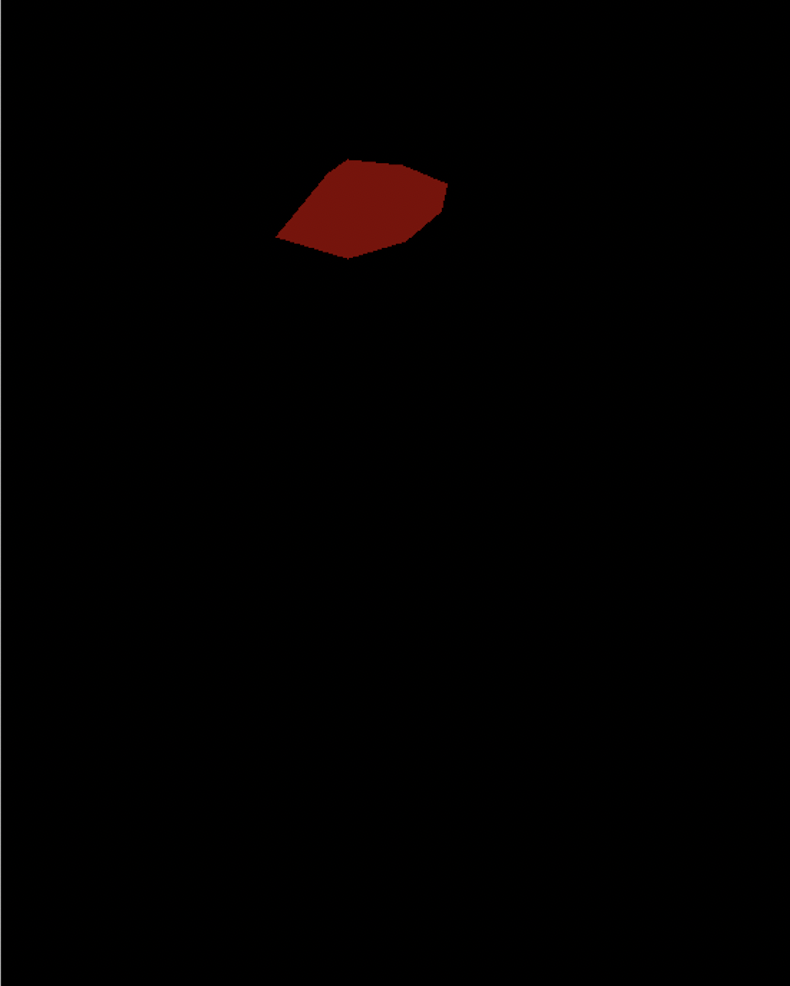
**

**Supplementary figure 2.** The CEUS frame and corresponding mask in both iu22 and Resona9 device.
